# Restoration of Retarded Influenza Virus-specific Immunoglobulin Class Switch in Aged Mice

**DOI:** 10.4172/2155-9899.1000403

**Published:** 2016-03-22

**Authors:** Yongxin Zhang, Ying Wang, Monica Zhang, Lin Liu, Innocent N Mbawuike

**Affiliations:** 1 ZYX Biotech Company, 1452 Halsey Way, Suite 100, Carrollton, TX 75007, USA; 2 Influenza Research Center, Department of Molecular Virology and Microbiology, Baylor College of Medicine, Houston, TX 77030, USA

## Abstract

**Objective:**

The declined immune response to infection causes significant higher morbidity and mortality in aging in spite of the coexisted hyperimmunoglobulinemia (HIG). This study is to reveal the cellular basis of HIG and mechanism of weakened HA-specific IgG response in aged mice and to test cell therapy in the treatment of age-related IgG antibody production deficiency with immunocyte adoptive transfer.

**Methods:**

BALB/c mice was immunized with Influenza A/Taiwan vaccine and challenged with the same strain of virus. ELISA was used to assess the levels of total immunoglobulins and antigen specific antibody response. The flow cytometry and ELISPOT were used to evaluate the frequencies of total immunoglobulin- and specific antibody-producing and secreting B lymphocytes. *In vitro* expanded mononuclear cells, CD4+ T lymphocytes and CD20+ B lymphocytes from old and young mice were adoptively transferred into influenza virus-challenged aged mice, and HA-specific IgG responses were observed.

**Results:**

It is found that old mice exhibited higher levels of total serum IgG, IgM and IgA, higher frequencies of IgG+, IgM+ and IgA+ cells, and greater antigen-specific IgM and IgA responses to influenza infection, in comparison to young mice. However, influenza antigen- specific IgG and its subclass responses in old mice were significantly lower.

**Conclusion:**

The retarded specific IgG response could be attributed to an insufficiency of immunoglobulin class switch in aging. Correlation analysis indicated that HIG and deficient specific IgG production in aged mice could be independent to each other in their pathogenesis. Correction of deficient specific IgG production by adoptive transfer of *in vitro* expanded and unexpanded CD4+ cells from immunized young mice suggests the CD4+ cell dysfunction contributes to the insufficiency of immunoglobulin class switch in aged mice. The transfusion of *in vitro* expanded lymphocytes could be a potential effective therapy for the age-related immunodeficiency and could play a role in the infection prevention in aging.

## Introduction

Some hyperimmunoglobulinemia (HIG)-related diseases, such as Waldenstrom’s macroglobulinemia, angioimmunoblastic lymphadenopthy, multiple myeloma, amyloidosis and certain autoimmune diseases, occur in old people much more frequently than in the young [1–4]. However, such a high immunoglobulin (Ig) tendency does not seem to promote the immunity against infections in old people. Some respiratory infectious diseases, such as influenza and RSV infection, have significantly higher morbidity and mortality in aging [5–10]. More than 90% of deaths caused by influenza occurred in old people [11,12]. It has been well demonstrated in both clinical and animal experiments that both cellular and humoral immunity to influenza virus and RSV infection decline in aging [13–21]. To determine whether HIG in aging is accompanied by an alteration of antigen-specific antibody production, we examined influenza virus antigen-specific and –nonspecific IgG, IgM and IgA and assessed IgG^+^, IgM^+^ and IgA^+^ B cells with ELISA, ELISPOT assay and/or flow cytometry in old mice and young controls.

The weakened antigen-specific humoral immune responses in aging involve the mechanism in which insufficiency of Ig class switch recombination (CSR) could play an important role. This may be resulted from the reduced T helper (CD4^+^) cells and the diminished germinal center reaction [22,23]. Many factors, such as CD40, CD40L, DNase I, and cytokines, affect or regulate CSR. Among these factors, some cytokines, such as IFN-γ [24–27], IL-4 [28] and TFG-β [29] have been demonstrated to be able to direct CSR from IgM to certain Ig class or subclasses. In this study, the total Igs levels in aged mice would be assessed in comparison with young mice to show the existence of HIG in aged mice. Second, IgG^−^ and IgM^−^producing and -secreting cells would be assessed using Flow cytometry and ELISPOT to elicit the cellular basis of HIG. Third, the productions of influenza virus-specific Igs would be observed kinetically in both young and old mice to demonstrate that the aged mice with HIG have a deficiency in the production of antigen-specific IgG. Fourth, the antigen specific IgG/IgM ratios at different time points would be calculated to reveal the obstacle of Ig class switch from IgM to IgG, which is involved in the deficiency of antigen-specific IgG production in elderly mice. Fifth, antigen-specific IgG^−^ and IgM^−^ prodicing and secreting cells would be assessed using purified IgM^+^, IgG^+^, IgM^−^, IgG^−^ and unsorted cell to elicit the cellular basis of the retarded CSR in aged mice. Sixth, the CSR regulatory role of CD4^+^ T cells in aged mice would be investigated by the adoptive transfer of purified CD4^+^ cells. The last, the *in vitro* expanded and unexpanded mononuclear cells, CD4^+^ T cells and CD20^+^ cells from young and old mice would be adoptively transferred into primed aged mice and the HA-specific IgG responses would be observed to explore the possibility to enhance the antigen-specific IgG responses in aged mice with the deficient CSR.

## Methods

### Mice and influenza virus infection and vaccination

Old (22–24 months) and young (4 months) BALB/c (H-2d) mice were purchased from Charles River Laboratories under a contractual arrangement with the National Institute on Aging. These animals were housed in specific pathogen-free certified rooms and cages were covered with barrier filters with sentinel cages for monitoring infections. The Baylor Animal Protocol and Research Committee approved the use of animals according to principles expressed in the National Institutes of Health, USPHS, *Guide for the Care and Use of Laboratory Animals*. The 16 old mice were divided into two groups (8 in each group), primed with 10× MID^50^ of influenza A/Taiwan/1/86 (H1N1) virus (FV) [17,30– 34] or with control MEM media by intranasal inoculation. The same was done for the 16 young mice, so that there were 4 groups total. Two months later, 4 mice in each group were challenged with the same dosage of FV by the same administration methods as priming. 10 additional mice (5 old and 5 young mice) were subcutaneously vaccinated with formalin- inactivated FV in 5 µg/20 µl each animal twice with the same interval as the live virus infection, which was demonstrated that animals could immunized well and have significant antibody responses. With the same priming and challenging strategies, the additional mice each group) were used for influenza antigen-specific ELISPOT assay. 12th day following challenging, bone marrow cells and splenocytes were obtained as described previously [23]. HA-specific IgG and IgM ELISPOT assay were performed with the mice two weeks after primary infection with the same dosage of virus infection. The cell isolation and expansion of lymphocytes from the peripheral blood 1000 µl (200 µl of each) of five immunized young mice were further performed in the biomedical laboratory of Zyxell Inc. and the cells were adoptively transferred into aged animals.

In the cell adoptive transfer study, 60 additional old mice were immunized with influenza vaccine (formalin-inactivated FV) and challenged with the live virus using same strategies as described above. Eight weeks after the initial immunization, each 20 of these aged mice received adoptive transfer of B-cells, CD4^+^ T cells or unsorted cells. Each of the 20 mice were further divided into five groups (4 in each group) and each group received the expanded cells from young mice or old mice, unexpanded cells from young mice or old mice, or 0.9% NaCl as control. The immunocyte donor mice, young and old, were immunized in the same way as described above eight weeks before the cells were taken.

### Lymphocyte cell isolation and expansion

The peripheral blood of five influenza virus-immunized young mice was drawn by retroorbital bleeding and the mononuclear cells were isolated using Lympholyte-M (Cedarlane) by following manufacturer’s instruction. Mononuclear cells were seeded at 2 × 10^5^ cells/mL in ZYX Bioreactor with IMDM supplemented with 10% ultra low IgG FBS containing 5.5 µg/mL transferrin, 10 µg/mL insulin, 6.7 ng/mL sodium selenite, 100 µg/ml streptomycin, 100 U/ml penicillin G (all from Invitrogen, Burlington, ON, Canada) and a mix of cytokines, namely 8 ng/mL IL-2 (80 U/mL), 40 ng/mL IL-10 (20 U/mL) (both from PeproTech, Rocky Hill, NJ, USA) and 100 U/mL IL-4 (R&D Systems, Minneapolis, MN, USA). Cultures were fed by replacing at least half of the culture medium every 3 days for total 12 days. CD4^+^ T cells and CD20^+^ B cells were then separated with AutoMACS (Miltenyi Biotec) and its corresponding magnet-antibody coupling reagent by following manufacturer’s instruction. Both T and B cell purities are greater than 97%, which was assessed by Flow cytometry with standard procedure (see following section).

10^7^ expanded cells and 5 × 10^5^ unexpanded cells were respectively injected into pre- immunized old mice through tail vein on the same day but 4 hours before animals were challenged with virus. Before injection, all cells (including unexpanded cells) were washed once with above expansion media, then washed 3 times with IMDM and re-suspended in 0.9% NaCl.

### Frequency determination of B cell subclasses using flow cytometry

2, 4 and 6 days following both priming and challenging with influenza virus, the peripheral blood lymphocytes were isolated by gradient with Lympholyte (Cedarlanen, Cat#CL5030). These cells and the isolated CD4^+^ T cells and CD 20^+^ B cells were stained with a rat monoclonal antibody specific for mouse IgG, IgM, IgA, CD138, CD19, CD 20 or/and CD4 conjugated to PE or FITC. Stained cells were stored at 4°C in the dark and analyzed within 24 hours using two-color flow cytometry (Beckman Coulter, Miami, FL, USA).

### Detection of antigen-specific and -nonspecific Igs-secreting lymphocytes using ELISPOTs

Combined antigen-specific IgG and IgM ELISPOTS assay were performed as described previously [23]. The fresh peripheral lymphocytes, bone marrow cells and splenocytes from primed old and young mice were directly assayed. Also, IgM^+^ cells, isolated from the spleens of these mice and purified with anti-IgM magnet beads and AutoMacs (Miltenyi Biotec Inc. Auburn, CA), were used to evaluate the efficiency of Ig class switch in old mice by ELISPOT assay. In this experiment, the purified IgM^+^ cells were cultured with the irradiated (3000 rads) influenza virus A/Taiwain virus infected splenocytes, mouse IL-4 (200 ng/ml) and Interferon-γ (100 ng/ml) (Bio-Rad) and assessed prior to and postculture. In the ELISPOT assay, nitrocellulose filters were coated with influenza A/Taiwan virus HA antigen at 4°C overnight, and washed three times with PBS, then added to 20 ml of blocking solution and incubated again at 4°C overnight. The filters were washed with PBS three times again and add RBC-free cells at 2 × 10^5^ cells/well to 96-well U plate (150 µl /well). The plates were covered with nitrocellulose sheet coated with Ag and paper tower, and turned upside down and the content of the wells was delivered onto the nitrocellulose filter by doing a one arm windmill spin; Plates were incubated at 37°C, 5% CO_2_ for 2 hours. The cells on membrane were then lysed with distill water for 15 min. After washing, the membranes were incubated at RT in blocking solution for 30 min. Following three times of wash, 1:1,000 diluted goat anti-mouse lgM-AP (Southern Biotechnology) and goat anti- mouse IgG-HRP (Southern Biotechnology) were added and the membranes were incubated at RT for 1 hour. AP and HRP activities were visualized using 3-amino-9-ethylcarbazole and napthol AS-MX phosphate/Fast Blue BB, respectively.

ELISPOTS assays to detect antigen-nonspecific Ig-secreting cells were carried out In the same way as did antigen-specific IgG and IgM ELISPOTS assays with following exceptions: rabbit anti-mouse Igs were used for the coating, HRP-labeled rabbit anti-mouse Igs for detection, only AEC substrate set (BD Pharmingen) for spots development and all reactions finished in MultiScreen 96-well Filtration Plates. Plates were read and reported by C.T.L. Analyzers LLC (Cleveland, OH).

### ELISA

Standard procedure was used in ELISA as we described before [17,19,35,36]. For the total IgG, IgM and IgA detection, the heavy chain-specific rabbit anti-mouse antibodies (5 µg/ml) were used for coating and the serum were 1:5 × 10^5^ diluted in Superblock. For antigen-specific antibody detection, influenza A/Taiwan virus HA antigen (5 µg/ml) was used for coating and Superblock 1:5 × 10^3^ diluted sera or 1:10 diluted culture supernatants were used as sample. All detection antibodies were Biotin-labeled heavy chain-specific or IgG subclass-specific rabbit anti-mouse antibodies (ZYMED Laboratories Inc. South San Francisco, CA). Plates were coated at 4°C overnight and washed with PBS-tween 20 four times. Each well was blocked with 200 µl Superblock (Pierce Biotechnology, Inc., Rockford, IL) at 37°C for 2 hours. Plates were then washed as before, 100 µl of diluted serum was added into wells in duplicates, and incubated at 37°C for 2 hours. Plates were washed again and 100 µl biotin-labeled detection antibody was added and incubated at 37°C for 1 hour. After washing, 100 µl of HRPO-streptoavidin was added to each well with incubation at 37°C for 0.5 hours. Then plates were washed 6 times and substrate (ABTS) was added to each well. After incubation at room temperature for 15 minutes, the plates were read at 405 nm with ELISA reader, UVmax kinetic Microplate Reader (Molecular Devices Corp., Palo Alto, CA). Results were extrapolated from the standard curve and were shown in ng/ml for total Ig determination or in U/ml for specific antibody determination with the software “SoftMax Pro” from the same company. The standards for total Ig determination were commercial purified IgG, IgM and IgA (ZYMED Laboratories Inc. South San Francisco, CA). The standards for the detection of antigen-specific antibodies were the frozen aliquots of combined positive mouse sera, and the highest titer was arbitrarily defined as 4000 U/ml. The pooled serum from negative control animals at different time points also served as experimental negative controls. All experimental negative controls were below the arbitrarily baseline (5 U/ml), which also served as quality control.

### Antigen- specific Immunoglobulin class switch assay

Splenocytes from primed old and young mice were sorted using magnet beads- labeled anti-mouse IgM and anti-mouse IgG (Miltenyi Biotec Inc. Auburn, CA) with AutoMax cell sorting machine (Miltenyi Biotec Inc. Auburn, CA) for the isolation of IgM^+^ cells and the depletion of IgG^+^ cells, respectively, by following manufacturer’s instruction. In brief, cells were blocked with Fc block antibodies at 4°C for 30 minutes, washed three times with running buffer (5% FBS and 0.2 mM EDTA in 1× PBS), and incubated with magnet- labeled antibodies (20 µl/10^7^ cells in 100 µl total volume) at 4°C for 30 minutes followed by three washes as before. Cells were sorted by cell sorting machine, and the purities of sorted cells, which were evaluated with flow cytometry, were equal to or greater than 98%. IgM^+^ cells, IgM^−^ cells, IgG^+^ cells, IgG^−^ cells and unsorted cells were cultured each with IL-4 (20 ng/ml) and the stimulator cells (splenocytes infected with influenza A/Taiwan virus for 4 hours and irradiated 3000 Rads of 6°C). Prior to and after three days of culture, different phenotypes of cells were respectively examined for IgG and IgM secreting cells with HA-specific ELISPOT assay, and HA-specific IgG and IgM in supernatant of cell culture on day 3 following the culture were assessed with ELISA as described above.

### Data analysis

Comparisons of differences among mean antibody levels, mean frequencies of Ig positive cells and mean number of Ig-secreting spots of different groups were made using the ANOVA procedure (STATVIEW Software, SAS Institute, Inc, Cary, NC, USA). Correlation analysis between total IgG and antigen-specific IgG were performed using Z test (STATVIEW Software, SAS Institute, Inc, Cary, NC, USA) [17]. A difference between comparison groups of P<0.05 level was considered significant.

## Results

### Elevated level of Igs in old mice

**Figure 1:**
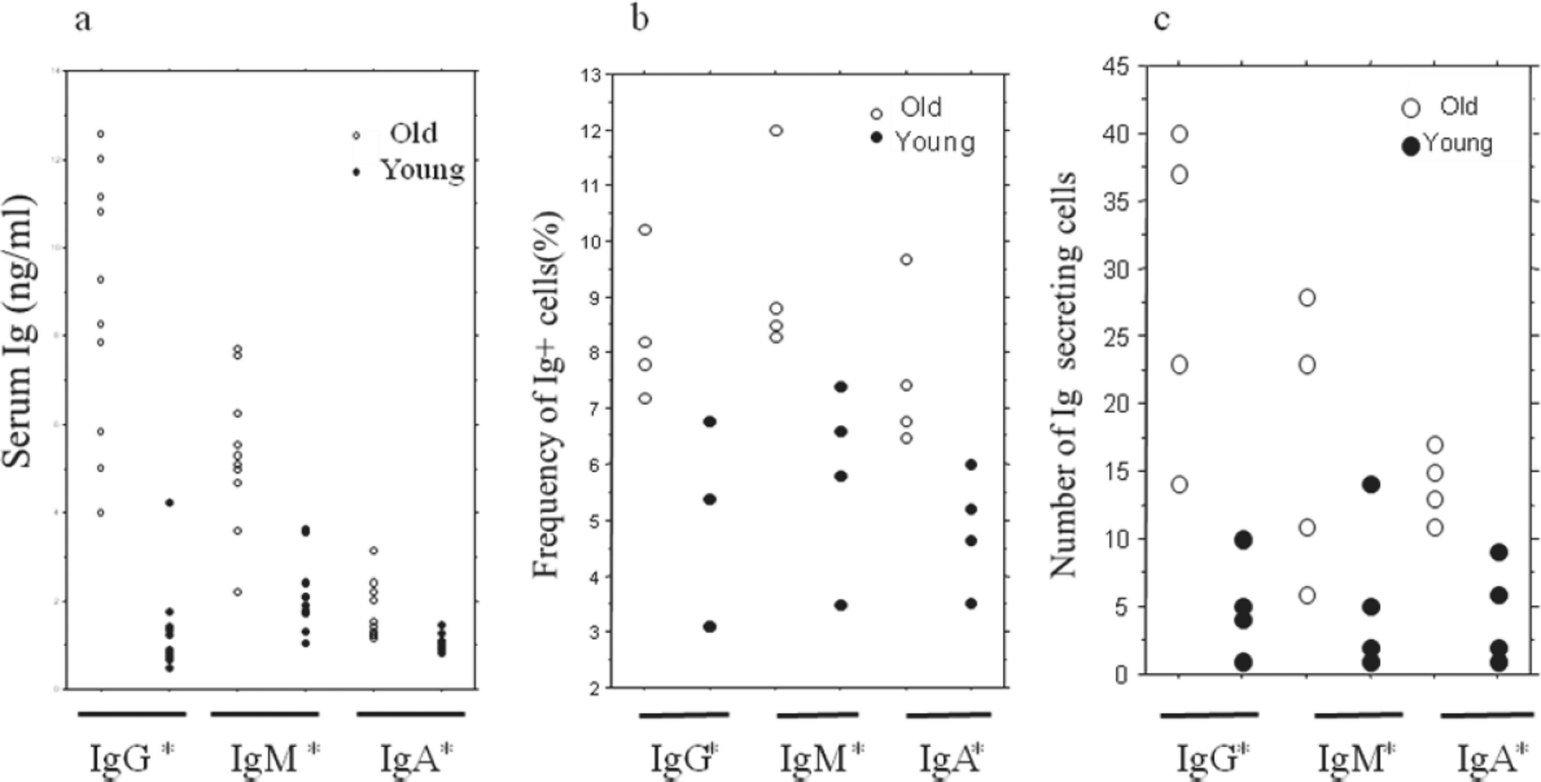
Serum total immunoglobulin levels (1a) and Ig-producing (1b) and Ig-secreting (1c) cells in old and young mice. 10 old (24 months of age) and 10 young (4 months of age) were bled and the 1: 5 × 10^5^-diluted sera were assessed for the 19 levels of total Immunoglobulin, IgG, IgM and IgA with ELISA **(1a)**. Compared to young mice, old mice exhibited significantly elevated levels of serum IgG (P<0.001), IgM (P<0.05) and IgA (P<0.0001). Four mice in each group were also bled for assessment of the frequencies and numbers of total Immunoglobulin IgG, IgM and IgA positive cells with Flow Cytometer **(1b)** and ELISPOT assay **(1c)**. For Flow Cytometry analysis, 0.4 × 10^6^ cells in each tube were stained with FITC- labeled rat anti-mouse Mab. For ELISPOT assay, 8 × 10^4^ cells in 0.2 ml were equally distributed into two wells and the ELISPOT plates were scanned and analyzed by C.T.L Analyzers LLC (Cleveland, OH). Compared to young mice, old mice exhibited significantly increased frequencies and spot numbers of IgG (P<0.01–0.001), IgM (P<0.05–0.01) and IgA (P<0.05–0.01) positive cells. The data were presented in means ± SE. Compare the young and the old mice, ^*^P<0.05–0.0001.

Eight old and eight young mice were bled for serum total immunoglobulin detection with ELISA. Old mice exhibited significantly higher levels of serum IgG, IgM and IgA (Figure 1a) compared to the young mice.

### The cellular basis of HIG in aged mice

As shown in figure 1b, the frequencies of IgG, IgM and IgA positive cells from peripheral blood of the old mice are all significantly higher than those of the young mice (P<0.05 or 0.01). Consistent with the flow cytometric data and elevated Ig levels in aged mice, Ig ELISPOT on peripheral lymphocytes also showed the significant increases of IgG-, IgM- and IgA-secreting cells in old mice compared to the young mice (Figure 1c).

### Reduced antibody response to primary and secondary influenza virus infection in old mice is selective for IgG

**Figure 2:**
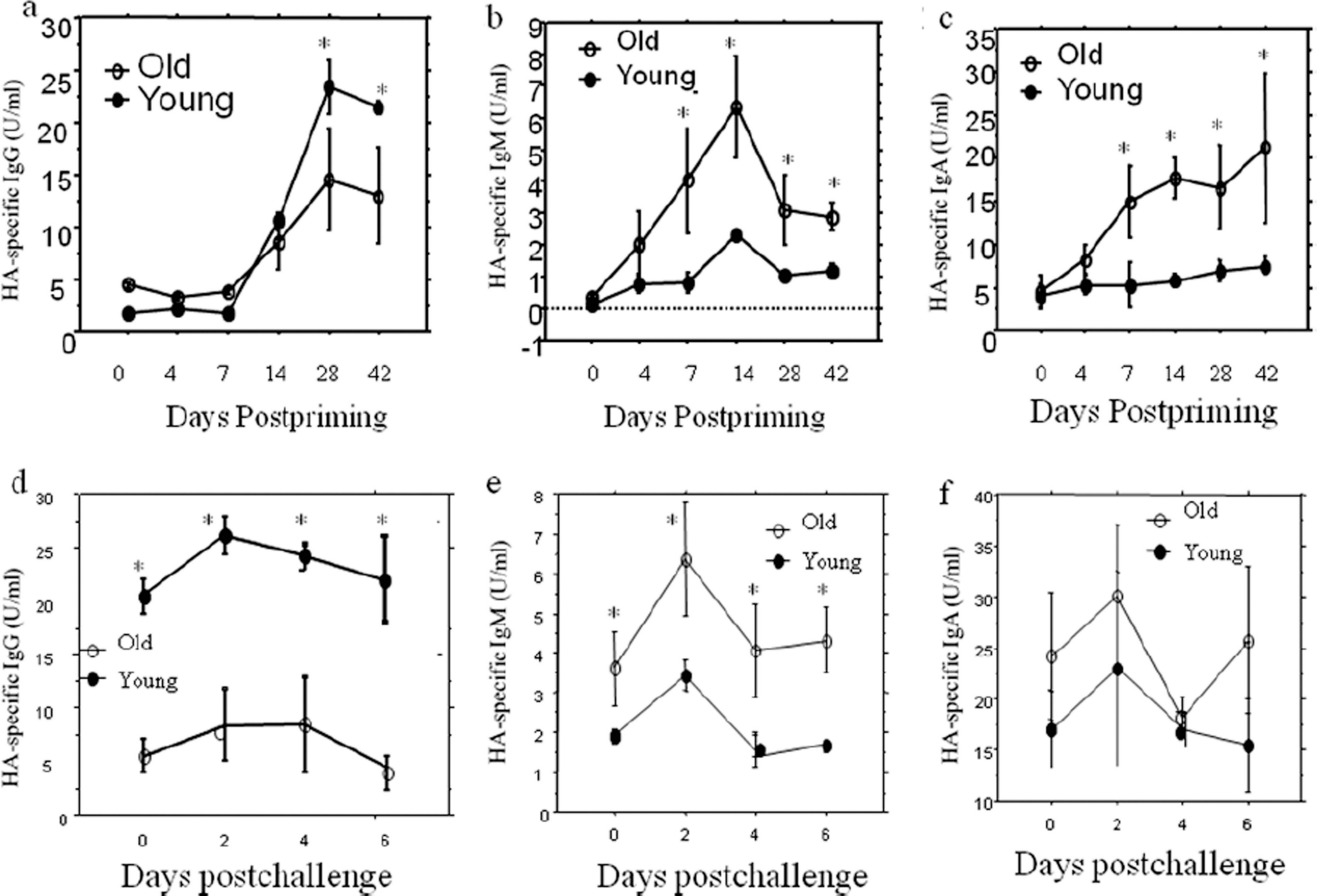
Reduced antibody response to primary (priming) and secondary (challenging) influenza virus infection in old mice is selective for IgG. Serum samples were collected from mice prior to and at various time points post-priming and post-challenging with 10 MID^50^ of influenza A/Taiwan/1/86 (H1N1) virus, respectively. HA-specific IgG, IgM, IgA were measured using ELISA. IgG antibody response to the influenza virus infection were lower in old mice than in young mice. The difference became significant (P<0.05) on the 28^th^ day after the primary infection **(2a)** and all time points for the secondary infection (P<0.001) **(2d)**. In contrast, the antigen specific IgM and IgA responses were all higher in old mice than in young mice (P<0.05 and 0.01) (2b, 2c, 2e and 2f) beginning on the 7^th^ day following priming with virus. Compare the young and the old mice at corresponding time points, ^*^P<0.05–0.001.

**Figure 3:**
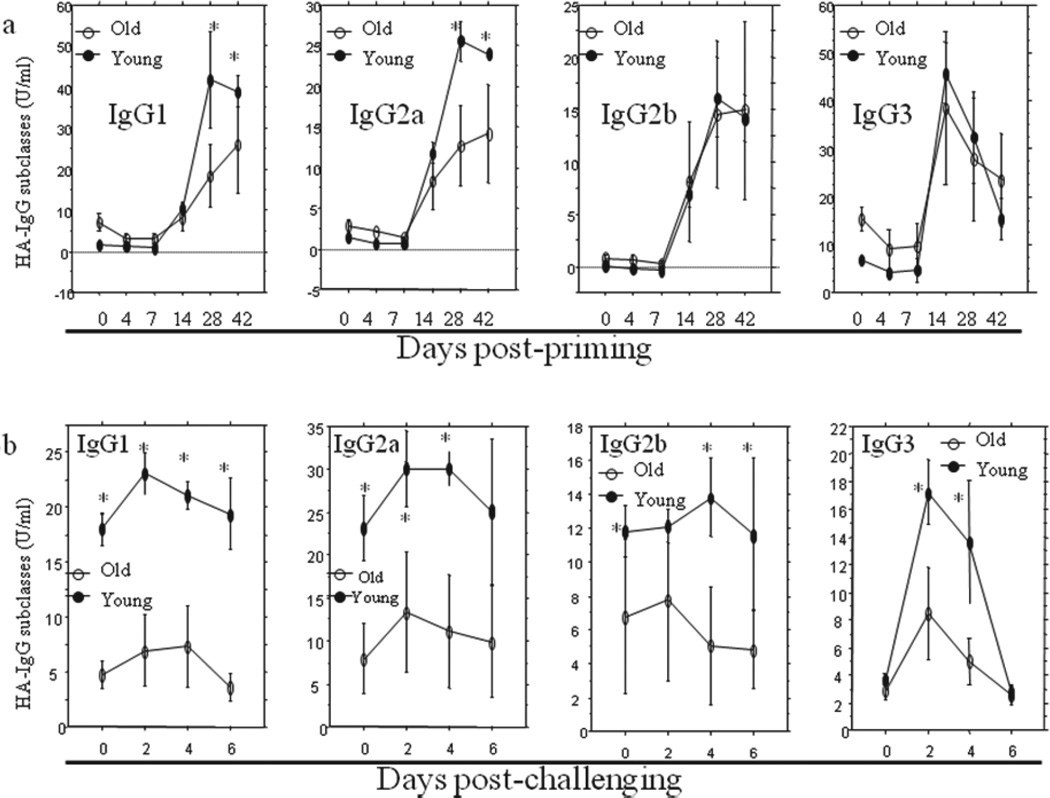
Influenza virus-specific IgG subclasses responses to primary (priming) and secondary (challenging) infection in old and young mice. Serum samples were collected from mice prior to and at various time points post-priming **(3a)** and post-challenging (3b) with 10 MID50 of influenza A/Taiwan/1/86 (H1N1) virus. IgG subclasses (IgG1, IgG2a, IgG2b, IgG3) were determined using ELISA. The levels of the antigen-specific IgG1 and IgG2a were at significantly lower levels in the old mice after day 28 post-priming in comparison with the young mice (P<0.05) (3a). Such a difference was not found for IgG2b and IgG3 (3a). In contrast, the levels of the antigen-specific IgG1, IgG2a and IgG2b for the challenging were significantly lower at all time points in the old mice than those in the young mice (P<0.05–0.001) **(3b)**. Such a difference (P<0.001) was found on day 2 and day 4 but not day 0 and day 6 post-challenge for IgG3 (3b). The data were presented in means ± SE for 4 mice in each group. Compare the young and the old mice at corresponding time points, ^*^P<0.05–0.0001.

**Figure 4:**
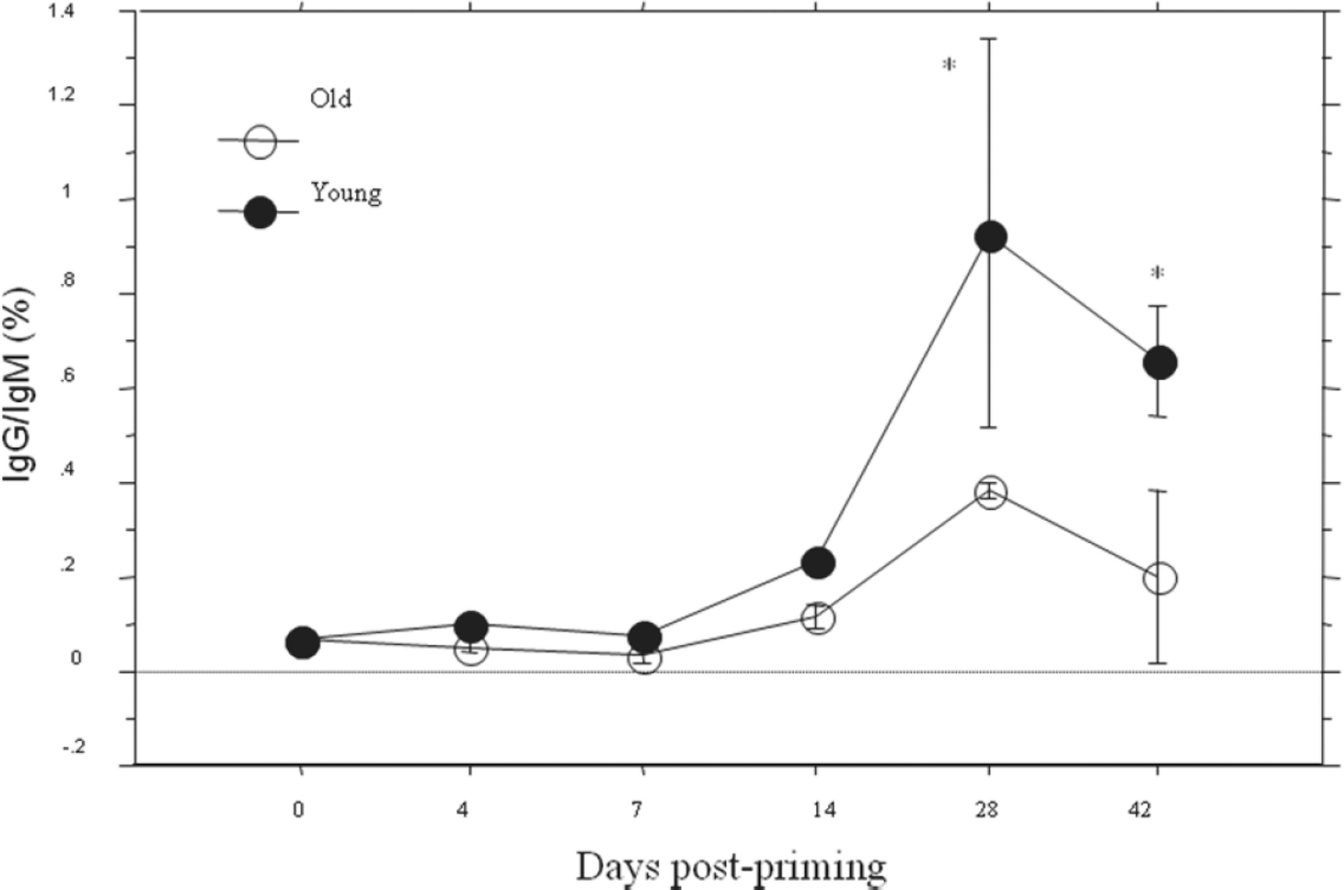
Kinetic change of serum IgG/IgM ratio. Data extrapolated from the same animals as above Figure 2. The curve of the IgG/IgM ratio was significantly lower in old mice than that in the young mice (P<0.01) postpriming. The data were presented in means ± SE. Compare the young and the old mice at corresponding time points, ^*^P<0.01.

Serum samples were collected from mice prior to and at different time points after infection with 10 MID50 of influenza A/Taiwan/1/86 (H1N1) virus. HA-specific IgG, IgM, IgA and IgG subclass (IgG1, IgG2a, IgG2b and IgG3) were measured using ELISA. The results showed that although serum total IgG level was higher in old mice, IgG antibody response to the primary influenza virus infection were lower in old mice than in young mice both after primary infection (postpriming, Figure 2a) and after secondary infection (post challenging, Figure 2d). Considering the sensitivity level (around 5 U/ml) of the assay, the IgG levels on day 0, Day 4 and day 7 are all at the baseline. The difference became significant (P<0.05) on the 28^th^ day after the primary infection (Figure 2a). In contrast, the antigen specific IgM and IgA responses were all higher in old mice than in young mice (P<0.05 and 0.01) both after primary infection (Figures 2b and 2c) and after secondary infection (Figures 2e and 2f), beginning on the 7^th^ day following priming with virus. Antigen-specific IgG subclasses analysis showed that the peak response levels of IgG1 and IgG2a following the primary infection (Figure 3a) and all IgG subclasses following secondary infection (Figure 3b) were all significantly lower (P<0.05–0.0001) than those seen in young mice and consistent with those seen for antigen-specific total IgG. IgG/IgM ratio was extrapolated from above antigen-specific IgG and IgM ELISA data. The curve for *in vivo* IgG/IgM ratio (Figure 4) kinetically exhibited the impaired capacity of Ig class switch in aging [23,37]. And such a deficiency in aged mice seemed only to exist in the switch from IgM to IgG (including IgG1, IgG2a and IgG2b) but not from IgM to IgA.

### Cellular evidences for the impaired capacity of Ig class switch in aged mice

**Figure 5:**
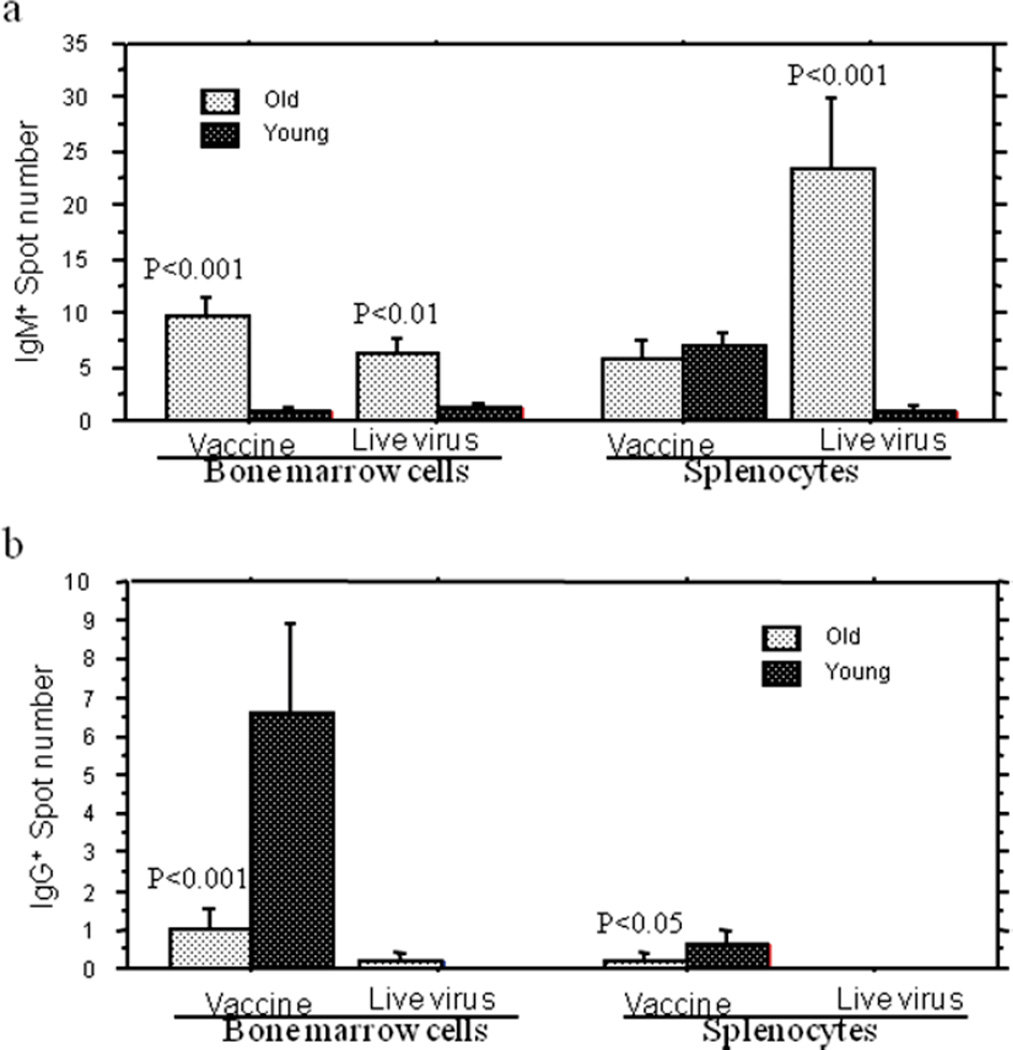
ELISPOT analysis for influenza virus-specific IgG^+^ and IgM^+^ cells in old and young mice. Bone marrow cells and splenocytes of old (24 months of age) and young (4 months of age) were harvested 12^th^ day after secondary infection (challenge) with 10 MID50 of influenza A/Taiwan/1/86 virus (FV) or after the second vaccination with inactivated FV. Cells were assessed for the numbers of influenza virus-specific IgM **(5a)** and IgG **(5b)** positive cells with two-color ELISPOT assay. Compared to young mice, inactivated virus- vaccinated old mice exhibited significantly decreased number of IgG (P<0.05–0.001) positive spots **(5b)**. Only one spot was found in live virus infection groups. IgM positive spots **(5a)** in bone marrow cells from both live virus-infected and inactivated virus-vaccinated old mice and in splenic cells from live virus-infected mice were all significantly increased when compared to those from young mice (P<0.01–0.001). The data were presented in means SE for 5 mice in each group.

Since very few positive antigen-specific spots (1–2 spots in each 96-well plate) were observed for peripheral lymphocyte samples, we used splenocytes and bone marrow cells for antigen-specific ELISPOT assay to evaluate the capacity of Ig class switch in aged mice. 12 days after secondary infection, the splenocytes and bone marrow cells were harvested from old and young mice immunized with either live influenza virus or inactivated virus vaccine. Two-color ELISPOT was performed for the determination of influenza virus antigen-specific IgG- and IgM- expressive cells. The data showed a significantly fewer specific IgG-secreting cells in spleen and bone marrow of aged mice compared to the young mice immunized with the same dosage of inactivated virus vaccine, but no significant differences were found with live virus (Figure 5b) because only 1 spot appeared in the test for live virus. In contrast, old mice exhibited significantly increased influenza virus antigen-specific IgM^+^ cells in spleen and bone marrow for live virus infection and in bone marrow for inactivated virus vaccination (Figure 5a). These data at cellular level further confirmed that the deficiency in Ig class switch contribute to diminished humoral immunity to influenza virus infection in aging.

### Insufficient HA-specific Ig class switch from IgM to IgG positive cells in aged mice

**Figure 6:**
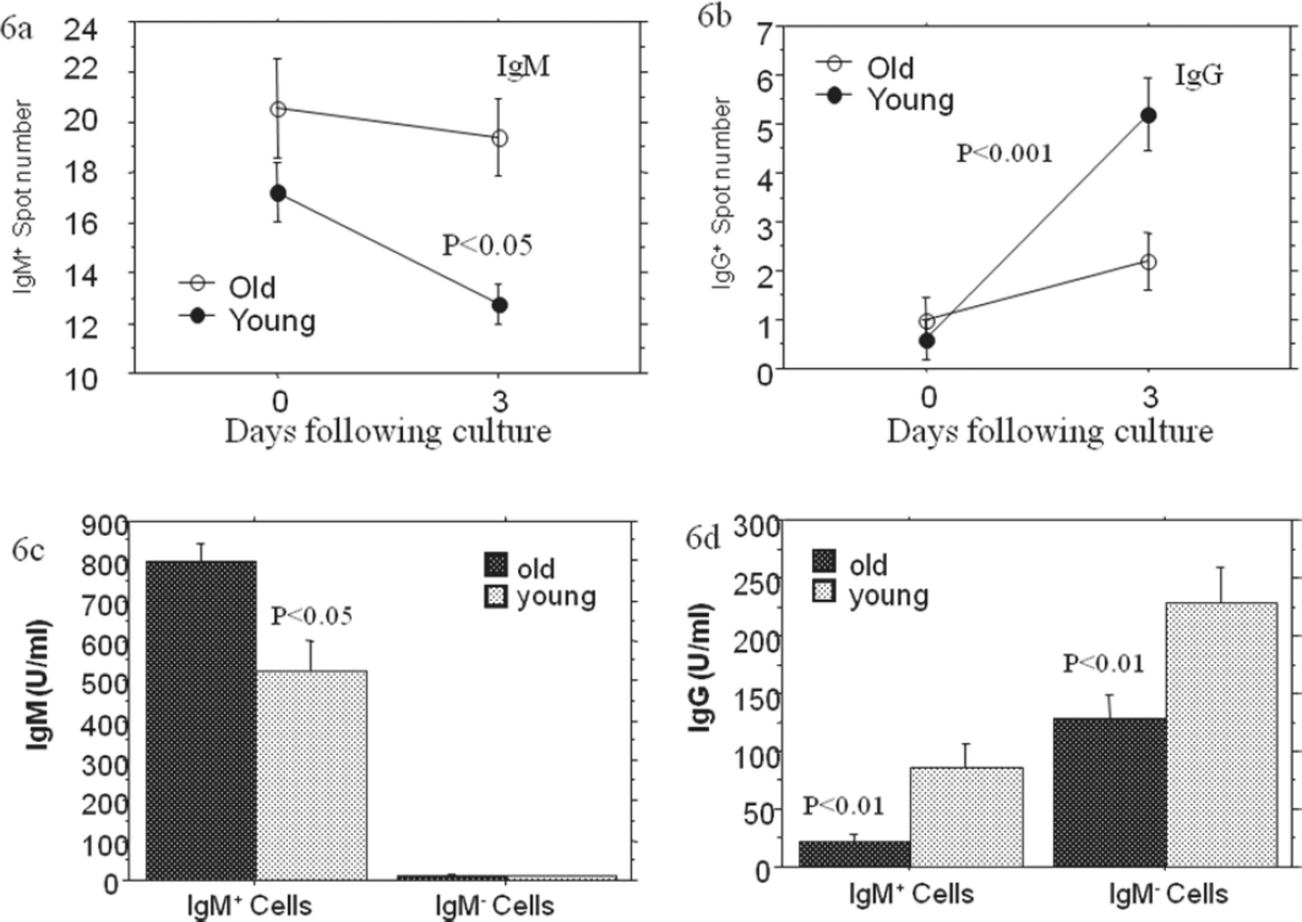
HA-specific IgG and IgM Production in purified IgM^+^ splenocyte culture. In experiment I **(6a and 6b)**, splenocytes were harvested from old (24 months of age) and young (4 months of age) two weeks after primary infection with the same dosage of influenza A/Taiwan/1/86 virus (FV) as in Figure 5 and IgM^+^ cells isolated with AutoMax system were cultured with IL-4 and 60Co-irradiated FV-infected splenocytes in an effecter/stimulator ratio of 5:1. ELISPOT assay was performed on the 1.6 × 10^6^ cells (in eight wells) prior to and day 3 following culture for IgM and IgG spots, respectively. The decreased mean IgM spot number and increased IgG spot number were exhibited from day 0 to day 3 in both old and young mice, but significant changes were only shown in young mice. The data were presented in means ± SE for 5 mice in each group. In Experiment II (6c and 6d), the supernatant of above culture on day 3 following culture were harvested and FV HA- specific IgM and IgG were measured with ELISA. The level of FV HA-specific IgM in the purified IgM^+^ cell culture of old mice is higher compared to the young mice, while the levels of FV HA-specific IgG in both purified IgM^+^ cell culture and IgM^+^ cell-depleted culture of old mice are significantly lower in the old mice compared to the young mice. The data were presented in means ± SE for 5 mice in each group.

In the immune response to antigen stimulation, IgM is the first antibody expressed on the antibody-producing cells. As the further stimulation of the same antigen and effects of cytokine (such as IL-4 and IFN-γ), most of IgM-producing cells switch to other Igs-, mainly IgG-producing cells, except for some that still express IgM after CSR. In order to further confirm the deficiency of Ig class switch from IgM to IgG positive cells in aged mice, and its effects on antigen-specific IgG antibody production, IgM^+^ cells, IgM− cells, IgG^+^ cells and IgG-cells, which were sorted with AutoMax cell sorting machine, as well as unsorted cells from old and young mice were respectively cultured with IL-4 and the irradiated influenza virus-infected cells, and the potency of these cells to produce HA-specific IgG was evaluated by antigen- specific ELISPOT assays. The purified IgM^+^ cells (Figures 6a and 6b) showed significantly less increase of HA-specific IgG spots and less or similar decrease of HA- specific IgM spots in elderly mice compared to the young mice after the three days of culture. These can also be seen in IgG-depleted (IgG−) cells (Data not shown for avoiding redundancy). As controls, IgM-depleted (IgM−) cells and unsorted cells exhibited far smaller differences between the old and the young in spot number changing, even if old mice had more IgM spots during the culture (Figure 6a). When the supernatant from above cell culture was collected on day 3 for ELISA, a significantly higher level of HA-specific IgM production (Figure 6c) and a more than 70%- lower HA-specific IgG production (Figure 6d) were observed on IgM^+^ cell culture of old mice compared to the young mice. IgM- cells from old mice also produced lower level of HA-specific IgG, but only 40% lower than did young mice.

### Relationship between serum total IgG and HA-specific IgG in old and young mice

**Figure 7:**
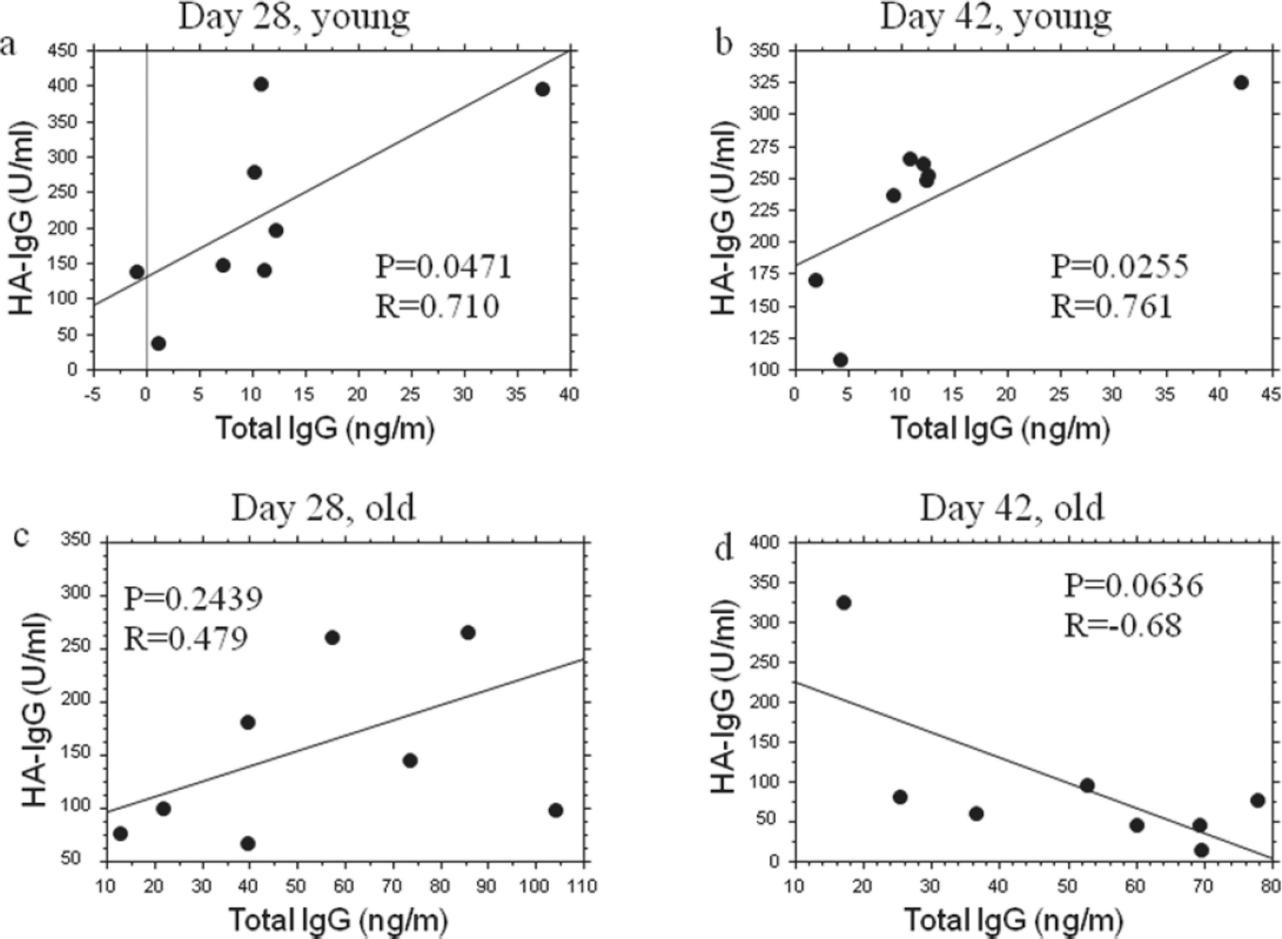
Relationships between total IgG and antigen-specific IgG for young and old mice. Total IgG and influenza A/Taiwan/1/86 virus (FV) HA-specific IgG were all measured with ELISA. The animals and infection approach are the same as above Figure 2. The young mice exhibited significant positive correlations between total IgG levels and FVHA-specific IgG levels on both day 28 and 42 after primary infection **(7a and 7b)** but old mice did not **(7c and 7d)**.

The serum from old and young mice on day 28 and day 42 after primary infection was assessed for total IgG and HA-specific IgG using ELISA. The young mice showed significant positive correlations between total IgG levels and antigen-specific IgG levels (Figures 7a and 7b) while old mice did not exhibit any significant correlations (Figures 7c and 7d).

### Correction of CSR deficiency in aged mice with CD4^+^ T cell adoptive transfer

**Figure 8:**
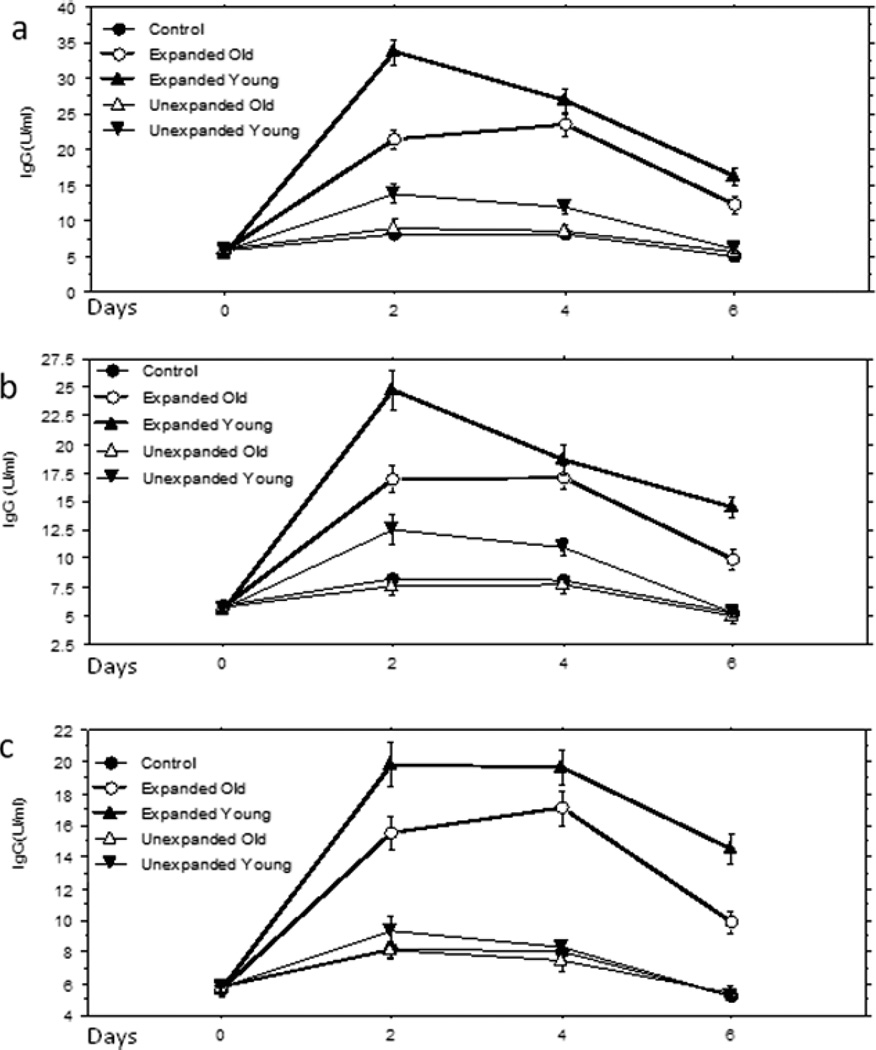
Elevated HA-specific IgG production by adoptive transfer in vitro expanded lymphocytes. 10^7^ expanded cells and 5 × 10^5^ unexpanded cells were respectively injected into pre-immunized old mice through tail vein. HA-specific IgG levels were assessed by ELISA in which the positive serum was used as a calibrator with arbitrary unit. The adoptive transfer with expanded mononuclear cells (Figure 8a), expanded CD4^+^ cells (Figure 8b) and expanded CD20^+^ B cells (Figure 8c) all elevated HA-specific IgG production (P<0.01 at days 2 and 4, P<0.05 at day 6). In contrast, only the unexpanded CD4^+^ T cells (Figure 8b) from young mice slightly increased the HA-specific IgG (P<0.05 at days 2 and 4 only). The mice received the expanded cells from young mice showed peak antibody response on day 2, which is significantly higher (P<0.01) than the peak response (day 4) of the mice received the expanded cells from old mice and significantly higher (P<0.01) than the antibody level of the mice received the expanded cells from old mice at the same time point (day 2).

Mononuclear cells from peripheral blood of immunized young and old mice were respectively equally divided into two parts. The first part was expanded in ZYX Bioreactor for 12 days with media containing IL2, IL4 and IL10. After the expansion, CD 20^+^ B cells and CD4^+^ T cells were isolated cells (the purity is greater than 95%, analyzed by flow cytometer) from half of total expanded mononuclear. Such 10 × 10^7^ expanded mononuclear cells, CD20^+^ B cells and CD4^+^ T cells were respectively injected into pre-immunized aged mice intravenously. The 2nd part was not expanded but was directly used to CD 20^+^ B cells and CD4^+^ T cells separation. These unexpanded cells (total mononuclear cells, CD20^+^ B cells and CD4^+^ T cells) were also adoptively transferred to pre-immunized aged mice. Four aged mice in each group received 1 × 10^7^ expanded cells (from 5 × 10^5^ unexpanded cells theoretically) per mouse or 5 × 10^5^ unexpanded cells per mouse. As shown in Figure 8a and 8b, the adoptive transfer of the expanded mononuclear cells and expanded CD4^+^ cells from both old and young mice and unexpanded CD4^+^ cells all significantly accelerated and elevated HA-specific IgG response to the virus challenge in aged recipients, while no changes was observed in the adoptive transfer of unexpanded CD4^+^ cells from old mice, in comparison with the control group in which mice did not receive cell adoptive transfer. In contrast (Figure 8c), with the same numbers of B cell adaptive transfer, only expanded cells significantly improved the specific IgG responses.

## Discussion

The serum Ig levels in healthy human in different age groups are variable. It was reported in 1970 [38] that most groups reached their peak Ig level around 40 years of age except for the black males who reached peak Ig level over 70 years of age. Another report in 1992 [39] showed a statistically significant age-related increase of the serum level of immunoglobulin classes (IgG and IgA but not IgM) and IgG subclasses (IgG1, 2 and 3, but not IgG4). Consistently in most reports, the largest extent of Ig concentration increase was shown in young children (<5 years of age [40]) and old people (>65 years of age). In our studies on IgG of the health adults, 10 middle age (20–40 years of age) and 10 elderly individuals (70–90 years of age), although IgG mean level in aged people is only slightly higher than that in the middle age, the difference is not so obvious as in our observation on mice. Considering the difference of nutritional condition between old and young people, serum albumin was used to normalize the IgG level. The normalized data showed that the ratio of IgG/albumin in elderly persons was significantly higher than that in the young. Similar to other reports, our data from normal people did not show an increased level of IgM in the elderly, which is different from our current observation on mice. Since in our observation more than 90% of mice over 24 months of age have enlarged spleen and it is quite common to find tumors in the spleen of old mice, and it was also reported that elderly people have higher morbidity of HIG-associated diseases (most of them are malignant disease) [1–3], the Ig level differences between old and young mice shown in this study could be a model for total human population rather than only for healthy people.

Clinically, the ranges for Ig normal value in elderly people and young adults are the same. Therefore, the Ig levels in the young mice in our current study can serve as normal controls for the evaluation of the Ig levels in aged mice. As shown in our experiment results, the old mice exhibited significant elevated total IgG, IgM and IgA levels. In contrast to the young, these old mice were obviously in HIG status. Such a HIG status in aged mice was further supported by the increased frequencies of Ig-producing cells (Flow Cytometry data, Figure 1b) and mean numbers of Ig-secreting cells (ELISPOT data, Figure 1c).

We then questioned whether the old mice with high level of total Ig could also produce adequate antigen-specific antibody in responses to new invading pathogen. We infected the old and the young mice with the same dosage of influenza virus, and used ELISA to kinetically monitor the changes of influenza A/Taiwan/1/86 virus (FV) HA- specific antibodies. Interestingly, among all main three Igs, only HA-specific IgG in old mice showed a significantly weakened response to the primary infection compared to the young mice, inconsistent with the total Ig levels, while both antigen-specific IgM and IgA showed enhanced responses, consist with their total Ig levels. To the secondary infection, the HA- specific IgG in old mice kept at lower levels and HA-specific IgM and IgA remained higher at almost all time points. When further analyzing HA-specific IgG subclass responses to the primary infection, it was found that both HA-specific IgG1 and IgG2 showed the same pattern as HA-specific IgG, but IgG2b and IgG3 did not show any significant differences between old and young mice, suggesting that the HA-specific IgG difference between old and young mice to the primary infection was mainly resulted from differences of IgG1 and IgG2a responses. To the secondary infection, however, all IgG subclasses in old mice showed lower levels of responses when compared with the young mice, which demonstrated the contributions of all IgG subclasses to the difference of HA-specific IgG responses between old and young mice. It was reported [25] that the aged mice had a decreased antigen-specific IgM and IgG2b production after influenza vaccination in addition to the weakened IgG1 and IgG2a. This disagreement with our data is possibly due to the differences of IgM response to different antigen preparations applied in different studies. Because the antigen-specific IgG is the main virus neutralization antibody [24–36], the decrease of its production can directly lead to the increase of morbidity and mortality due to infection in aging.

Studies *in vitro* have suggested that the compromised antigen-specific humoral immune responses in aging related to the insufficient Ig CSR [23,37]. The curve for IgG/IgM ratio (Figure 4) reflected efficiency of *in vivo* Ig class switch after primary FV infection and provided an evidence for the impaired capacity of Ig CSR in aged mice. The combined ELISPOT data from unsorted splenocytes and bone marrow cells revealed the relationship between IgM- and IgG- secreting cells in the same assay and demonstrated aged mice had significantly higher HA-specific IgM spots and lower HA-specific IgG spots in response to live virus infection or/and inactivated virus vaccination (Figure 5).

Diminished IgG antibody production in aging may be resulted from the reduced CD4^+^ T cell number and decreased CD4^+^ T cell function, the diminished germinal center reaction, and the defective bone marrow environment that has diminished ability to support the selection and survival of long-term Ab-forming cells [22,23,41–45]. In our previous studies [17], we have demonstrated that FV-primed old mice had a decreased splenic CD8^+^ and CD4^+^ T lymphcyte responses to FV stimulation in vitro. In current study, we repeated that experiment and examined peripheral CD4^+^ and CD8^+^ T cells responses. All data are very consistent. Combined these data, we agree the decreased CD4^+^ T cell levels could be a causative factor for the weakened antigen-specific humoral immune responses in aging. Many other factors, such as CD40, CD40L, DNase I, and cytokines, affect or regulate CSR. Among these factors, some cytokines, such as IFN-γ, IL-4 and TFG-β, have been demonstrated to be able to direct CSR from IgM to certain Ig class or subclasses [24–29]. IL-4 has been broadly demonstrated to be able to enhance CSR from IgM to IgG1 and IgE, and IFN-γ has a role to increase CSR from IgM to IgG2a and inhibition effect on IgA production [29]. Since our data indicated that the deficiency of antigen-specific humoral immune response in aged mice is selective for IgG, especially for IgG1 and IgG2a in primary infection, such deficiency is more likely caused by decreased cytokines, decreased expression of cytokine receptors on the B cell undergoing CSR and/or dysfunction of the signaling system of these cytokines. We have demonstrated that old mice have lower level of IFN-γ production [19], which might be the reason for the decreased IgG2a and increased IgA in aged mice. We did not find any significant difference in IL-4 production between old and young mice [19]. The evaluation of IFN-γ receptor and IL-4 receptor expression on antigen-activated IgM^+^ cells is ongoing.

In order to acquire a direct evidence of CSR deficiency form IgM to IgG in aging, which has not been shown in previous studies, we examined antigen-specific IgG production using purified IgM^+^ cell culture. After 3 days following the culture of the purified IgM^+^ splenocytes, the mean spot number for HA-specific IgG significantly increased in the young mice but not significantly in the old. Consistent to the ELISPOT data, ELISA performed with the supernatant from both IgM^+^ and IgM− cell culture (day 3) showed significant lower level of HA-specific IgG protein in aged mice compared to the young mice. Although IgM− cells (containing all IgG^+^ cells) produced higher levels IgG in both old and young mice than did IgM^+^ cells, the IgG production in young mice was more than three-fold as high as that in old mice for IgM^+^ cell culture while the IgG production of the young was only less than two-fold as high as that in old mice for IgM- cell culture. This fact also supports that deficient CSR from IgM to IgG contributes to the decreased antigen-specific IgG production in aged mice (Figure 6d).

The total IgG level is often used to evaluate patients’ immune competency in clinic [46]. Our data from old and young mice showed that total IgG levels and HA-specific IgG levels were in positive correlation in the young 28 and 42 days after primary infection (7a and 7b), but the old mice did not exhibit any significant correlation between total IgG levels and HA-specific IgG levels on these two days. This suggests that the response competency of antigen-specific IgG in the young mice can be reflected by their total IgG level but not in the old. The absence of significant correlation between total IgG levels and HA-specific IgG levels in aged mice also indicated that HIG was not the causative factor for the decreased antigen-specific IgG response and both HIG and deficient specific IgG production could be independent phenomenon in the senescence process of mice.

The adoptive transfer of CD4^+^ T cells has been used in many studies [47–50], especially on its regulatory effect on CD8^+^ T cells. In our current study, it was demonstrated that the decreased CD4^+^ T cell up-regulation to specific immune response in aging was reversed by the adoptive transfer of unexpanded mononuclear cells and CD4^+^ T cells from young mice and *in vitro* expanded mononuclear cells and expanded CD4^+^ T cells from both young and old mice. This further confirms that the deficiency of CD4^+^ T cell is at least one of the reasons for the declined specific immune response of aged mice, and revealed that *in vitro* expanded total mononuclear cells and CD4^+^ T cells from old donor can also function well. The latter provides a potential method to accelerate and enhance humoral immune response in clinic for aged people, in which the total mononuclear cells or CD4^+^ T cells from an aged individual can be expanded ex vivo and then adoptively transferred back. In current study, the expanded CD4^+^ T cells were significantly more potent in up-regulating antigen-specific IgG production than unexpanded CD4^+^ T cells, which was mainly resulted from the higher (20-fold more) cell number of the expanded cells but the role of cytokine stimulation *in vitro* should also be considered. Unlike CD4^+^ T cells, a small amount of adoptive transfer of CD20^+^ B cells from young mice could not significantly increase Influenza virus- specific antibody immune response. However, the ex vivo expanded CD20^+^ B cells from both old and young mice were not significantly affected by CD4^+^ T cells in old mouse body for their functioning, suggesting that effective dose of the *in vitro* expanded auto lymphocytes could be used to enhance the immune response in aging. It should be noticed that the cell number used in expanded cell adoptive transfer was much higher than that in unexpanded cell transfer and the former is almost equal to (slightly less than) the expanded amount of the latter, a small amount of expanded cell adoptive transfer could not increase the specific immune response in our pilot experiments. These indicated that the larger amount of lymphocyte adoptive transfer could help the aged mice to recover their immune response to the infection. Considering the possibility that the lymphocytes were activated by cytokines in vitro, it is much more effective and feasible in clinic to do the auto-transfusion with *in vitro* expanded large amount lymphocytes than with unexpanded large amount lymphocytes.

In summary, total serum Igs (IgG, IgM, IgA) were all at higher levels in old mice and increased Ig-producing and -secreting cells are the cellular basis of higher level of serum Igs in aged mice. In contrast to total Ig levels, influenza antigen-specific IgG antibody responses to influenza virus primary and secondary infection in aged mice were significantly lower than those in the young mice. All IgG subclasses were involved in the lowered IgG antibody responses. The deficiency in Ig class switch is at least one reason for the diminished humoral immunity to influenza virus infection in aging. HIG and deficient specific IgG production could be independent to each other in their pathogenesis. The diminished humoral immunity can be corrected by the adoptive transfer of *in vitro* expanded total mononuclear cells, expanded CD4^+^ T cells or expanded CD20^+^ B cells.
